# Supplementary material for: Adjuvant use of melatonin for pain management in endometriosis-associated pelvic pain—A randomized double-blinded, placebo-controlled trial
Source: PLoS One. 2023 Jun 2;18(6):e0286182. doi: 10.1371/journal.pone.0286182 (PMC10237656; doi:10.1371/journal.pone.0286182)
Supplement: S1 File — (DOCX) [file pone.0286182.s003.docx]

**Protocol in english**

**Effect of Melatonin on pelvic pain in patients with endometriosis**

**Bakground**

Endometriosis is a chronic progressive, inflammatory disease due to ectopic location of endometrium, estrogen dependent, causing cyclic and acyclic pelvic pain as well as symptoms from the intestinal and urinary tract and sometimes infertility. Dysmenorrhea is a common symptom. Several studies suggest that dysmenorrhoic women have a hyper sensitization of pain fibers leading to dysmenorrhea being classified as a Chronic pain syndrome .

Analgesics and/or hormonal therapy are most commonly used treatments for both dysmenorrhea and endometriosis.

Melatonin is mainly synthesized in Corpus Pineale, through hydroxylation from tryptophan to serotonin. Previous studies have shown effect of melatonin on acute and chronic pain and has anti-oxidative and anti-inflammatory properties which are relevant for both endometriosis and dysmenorrhea.

Reduction of pain in women with endometriosis has been shown at a dose of 10 mg melatonin daily. We chose a higher dose to evaluate if the reduction of pain could be more effective with a higher dose.

**Intervention**

20 mg melatonin or placebo given orally, at bedtime every day for 2 menstrual cycles or 2 months.

**Aim**

To explore if melatonin given daily has an analgetic effect in endometriosis associated pain, its tolerability. To explore if melatonin has a positive effect on sleep and quality of life.

**Primary outcome**

Pain evaluation according using VAS- visual analogue scale. A reduction of 13 mm is considered clinically significant.

**Secondary outcomes**

The use of other analgesics, paracetamol and/or ibuprofen.

Assessment of sleep with ISI- insomnia severity index

Assessment of quality of life with EHP 30 – Endometriosis health profile

Assessment of catastrophization with PCS – pain catastrophizing scale

Side effects of melatonin

Acceptance of melatonin

**Study design and method**

A randomized placebo-controlled, double-blinded, parallel trial, in which, after one observational cycle (4 weeks if amenorrhoic), participants are randomized to 20 mg melatonin/placebo taken at bedtime for 2 consecutive menstrual cycles (8 weeks if amenorrhoic). EHP 30 (endometriosis health profile), ISI (Insomnia severity index) and PCS (Pain catastrophizing scale) will be completed at the end of the observational period and treatment period respectively. The respondents will fill in a digital pain and bleeding diary daily. They will also be required to report what other analgesics they take daily. Adverse effects can be reported daily in the same digital diary.

**Criteria for inclusion**

Women, >17 yrs, with endometriosis (endometriomas or diagnosis at laparoscopy), severe pain (VAS >/= 4)

Good general health

Understands and speaks Swedish

If any other treatment, unchanged regimen for the last 3 months

Signed informed consent

**Criteria for exclusion**

History of disease in kidney or liver

Use of opioid analgesics

Smoker

Pregnant

**Population size**

Previous studies have shown that a reduction of 13 mm on VAS is of clinical significance . For an 80% power, level of significance 0.05, we need 15 respondents in each group. We plan to recruit a total of 40 to compensate for drop outs.

**Recruitment**

Through posters in the hospital and out-patient clinics. And via patient organization for endometriosis.

**Informed consent**

Potential participants will be informed verbally and in written by a nurse or a doctor in charge och the study. During the first visit the doctor in charge will procure signed document of informed consent upon enrollment.

**Statement of compliance**

The study will be conducted according to the protocol and current regulation, LVFS 2011:19, ICH GCP and the latest version of the declaration of Helsinki.

**Significant changes**Any significant changes to the protocol (LVFS 2011:19,7 kap) needs to be approved by the Swedish medical products agency and the regional board of ethics prior to implementation.

**Management of study drugs**

Packaging and labeling of study drug and placebo will be handled by APL (Apotek Produktion & Laboratorier).

**Randomization**

Will be done with a randomization computer program in blocks of 4.

**Analysis**

The study population will be analyzed with descriptive statistics. The differences in pain experience will be analyzed with mixed model method or repeated ANOVA. Sensitivity analysis will be made before locking the data base och unblinding the data.

**Adverse events (AE)**

An adverse event will be defined as any untoward medical occurrence in a subject without regard to the possibility of a causal relationship. Adverse events will be collected after the subject has provided consent and enrolled in the study. AE will be classified as mild, moderate or serious.

**Adverse reaction (AR)**

Every AE will be assessed regarding relatedness to the study drug.

**Serious adverse event (SAE)**

A serious adverse event for this study is any untoward medical occurrence that is believed by the investigators to be causally related to study-drug and results in any of the following: Life-threatening condition (that is, immediate risk of death); severe or permanent disability, hospitalization, or a significant medical hazard. Every SAE will be reported to the sponsor within 24 hours.

**Suspected Unexpected serious Adverse Reaction (SUSAR)**

A SAE probably related to the study drug and previously unknown will be reported to the board of ethics by mail within 7 days and to the Swedish medical products agency within 15 days.

**Risk-benefit**

The benefit if melatonin proves to be effective as an analgesic will be experienced as improvement of the participant’s condition. Which would be a welcomed addition to the treatment options. The participants can use their regular analgesics during the trial. Melatonin has not been associated with any serious adverse effects in previous trials.

**Criteria for termination**

The participants are free to terminate their participation at any point of the trial. The study responsibles can terminate the participation if e.g. a pregnancy occurs or if the safety of the participant cannot be secured. Or if a AE occurs negatively affecting the participant.

**Emergency unblinding**

In case of a SUSAR emergency unblinding will be made by the sponsor.

**Quality control**

CRF (case report form) are filled out by the nurses and doctors in charge of the study and stored safely. A research nurse from Linköping University will monitor the trial during the trial period.

**Source data**

CRF and medical files are treated as source data. As well as PCS, EHP 30, ISI. All source data will be archived securely for 10 years.

**Time plan**

Recruitment and start of the study is planned for November 2018 and is estimated to last 18 months. Analysis and processing of data is planned for 2020.

Protokoll studie:

Smärtlindring vid svår menssmärta och endometrios

Ansvariga:

Lena Marions docent överläkare KK Södersjukhuset
Lisa Söderman specialistläkare KK Södersjukhuset

Måns Edlund Specialistläkare Med dr ViforPharma AB

Ylva Böttiger Professor Överläkare Klin farmakologi, Linköpings universitet

LMS 2017:4 Eudra CT 2017-002287-40

2018-09-25

Innehållsförteckning sida

Bakgrund 9

Behandling med analgetika_____________________________________ 9__________

Hormonell behandling 9

Melatonin 10

Utvärdering av smärta 11

Dosval 11

Syfte 12

Frågeställningar 12

Primära utfallsvariabler 12

Sekundära utfallsvariabler 13

Design 13

Metod 13

Inklusionskriterier 14

Exklusionskriterier 14

Studiepopulations storlek 15

Urval 15

Informerat samtycke 16

Statement of compliance 16

Väsentlig ändring 16

Detaljerad plan för studiedeltagarna 16

Delstudie 1 (dysmenorre) 16

Delstudie 2 (endometrios) 17

Deltagarlogg 17

Behandling – studieläkemedel 17

Läkemedelshantering 17

Randomisering 18

Analys 18

Avvikande händelser (Adverse events=AE) 18

Avvikande reaktion (Adverse Reactions) 18

Allvarlig avvikande händelse (Serious adverse events=SAE) 18

Misstänkt oväntad SAR (SUSAR) 19

Risk/nytta värdering 19

Kriterier för avbrott i studien 19

Brytning av kodning 20

Kvalitetskontroll 20

Källdata 20

Tillgång till källdata 20

Förvaring av källdata 20

Tidsplan 20

Referenser 21

**Bakgrund:**

Dysmenorre är ett vanligt tillstånd bland unga kvinnor och beror på kontraktioner i uterus till följd av ökad produktion av prostaglandiner. Kontraktionerna leder till ischemi som i sin tur orsakar smärta. Förutom smärta kan även systemiska symtom som illamående, kräkningar, diarré, huvudvärk och syncope framkallas till följd av frisättning av ett flertal cytokiner. I tillägg till detta rapporterar många kvinnor att prestationen påverkas negativt såväl fysiskt som mentalt. Detta kan vara ett resultat av att sömnstörningar inte är ovanligt vid svår mensvärk liksom annan bäckensmärta.

Det är oklart hur vanligt det är med svår menssmärta bland unga kvinnor, tillgängliga data rapporterar allt mellan 8 och 33% . Mensvärk kan för många vara handikappande och medföra frånvaro från skola och andra aktiviteter.

Endometrios är ett östrogenberoende, inflammatoriskt tillstånd där endometriet är ektopiskt beläget, dvs utanför uterus och som kan orsaka kraftig smärta och också är associerat till infertilitet. Av de unga kvinnor som har svår mensvärk har omkring 75% faktiskt endometrios men diagnosen ställs i genomsnitt först 6.7 år efter symtomdebut. Kvinnor med endometrios har i flera studier uppvisat en försämring av upplevd livskvalitet.

Behandlingsprincipen av endometrios och menssmärta är densamma dvs att uppnå smärtfrihet. Smärtfrihet kan uppnås genom analgetika men det mest effektiva är att interferera med menstruationscykeln och/eller hämma ovulationen.

**Behandling med analgetika**

Den mest använda formen av smärtlindring är NSAID som hämmar syntesen av prostaglandiner. Det finns dock inte någon dokumenterad effekt på smärta vid endometrios. Tillägg av paracetamol kan ytterligare potentiera effekten. Nackdelen med dessa NSAID är risk för gastrointestinala besvär. För de som besväras av sömnstörningar till följd av smärta hjälper heller inte NSAID. En annan negativ effekt för kvinnor som önskar bli gravida är att NSAID preparat genom sin COX-hämmande effekt kan bidra till upphävd ovulation och därmed minskade chanser till graviditet. Det är en reversibel effekt men många kvinnor med endometrios har en aktuell graviditetsönskan och infertilitet är vanligare bland dessa kvinnor. Vid graviditet brukar dessutom besvären mildras påtagligt.

**Hormonell behandling**

Den hormonella behandlingen kan göras på olika sätt. Det enklaste är att använda hormonell antikonception som t.ex kombinerade p-piller eller liknande (ring/plåster). Enbart gestagen är också effektivt, lokalt i uterus för att minska blödningarna eller systembehandling för att hämma ovulationen. Dessa behandlingar kan också kombineras. I svårare fall krävs mer potenta läkemedel. GnRH analoger kan användas för att mer effektivt nedreglera ovarialaktiviteten men har ofta besvärande bieffekter till följd av låga östrogennivåer. I några fall måste också kirurgi användas då endometrioshärdar avlägsnas kirurgiskt.

En effektiv och tidigt insatt behandling kan förebygga mer avancerade former av endometrios. Det är därför viktigt att identifiera unga kvinnor med svår mensvärk för att tidigt kunna erbjuda effektiv behandling. Vi planerar därför att utvärdera den smärtlindrande effekten av melatonin hos kvinnor med dels svår mensvärk, dels konstaterad endometrios

**Melatonin**

Melatonin syntetiseras genom omvandling av tryptofan till serotonin och sker företrädesvis i corpus pineale (CP). Syntes sker även i ovarier. Frisättningen från CP sker framför allt nattetid när den elektriska aktiviteten i CNS (hypothalamus) är som lägst. Cirkulerande melatonin har en halveringstid på c:a 20-30 minuter och metaboliseras huvudsakligen i levern genom CYT P 450 oxygenering. Exogent melatonin administreras frikostigt i många länder till framför allt barn med sömnstörningar. Det finns idag ett flertal studier som visar att melatonin också har god effekt på smärta. Melatonin ökar frisättningen av b-endorfin och den smärtlindrande effekten har demonstrerats för såväl akut som kronisk smärta. Dysmenorre kan beskrivas som en akut smärta medan endometrios närmast är ett kroniskt smärttillstånd. Oxidativ stress anses vara en viktig faktor för uppkomsten av endometrios och just den antioxidativa effekten av melatonin vid endometrios har visats i djurstudier där endometrioslesioner minskat påtagligt (Yilmaz et al 2015). Melatonin har i en annan experimentell studie (Yildirim et al 2010) jämförts med aromatashämmaren letrozole som anses effektiv mot endometrios genom sin hämmande effekt på östrogenproduktionen. Man noterade där att melatoninbehandlingen resulterade i mer uttalad regression av endometriosförändringar än letrozole och dessutom medförde melatoninbehandlingen att förändringarna återkom i lägre utsträckning efter avslutad behandling. Studier har också visat att särskilda markörer för oxidativ stress signifikant påverkats av tillförseln av melatonin. Effekten har visats vara dosberoende där högre dos melatonin, 20 mg/kg/dag, minskade endometrioslesionerna i högre grad jämfört med den lägre dosen,10mg/kg/dag. Dessutom har djurstudier visat tydlig dos-beroende effekt på smärta av melatonin, oberoende av administrationssätt. Liknande effekter har man sett hos människa där doser på 0.05/mg/kg/dag, 0.15 mg/kg/dag samt 0.25mg/kg/dag jämfördes avseende effekt på smärta i testsituation (hög värme, tryck). Studien demonstrerade en tydlig korrelation mellan melatoninkoncentrationen och smärttröskeln hos försökspersonerna. En nyligen genomförd klinisk RCT kunde påvisa signifikant smärtlindring av daglig tillförsel av 10 mg melatonin i 8 veckor hos kvinnor med bäckensmärta till följd av endometrios. Fortfarande rapporterade dock kvinnorna bäckensmärta, även om de skattade den betydligt lägre. Att öka dosen till 20 mg vid just kronisk bäckensmärta har ännu inte studerats men experimentella studier stöder teorin att en högre dos kan behöva administreras vid endometrios (se nedan om dosval). Vi saknar dock exponeringsdata från djurstudier och har svårt att överföra dessa resultat till människa.

Preparatet är i Sverige registrerat på indikationen sömnstörning, en effekt som varit känd länge. Läkemedlet har också visats sig ha ångestlindrande effekt. Melatonin har få biverkningar och förefaller tolereras bättre än t.ex NSAID. Ett flertal studier har visat på att säkerheten med melatonin är god. Experimentella studier har inte visat några allvarliga sidoeffekter men då metaboliseringen av de flesta läkemedel hos t.ex möss sker betydligt snabbare än hos människa behöver ju detta också bekräftas också i humanstudier. Få eller inga sidoeffekter har dock noterats efter behandling med melatonin för sömnstörningar hos barn och vuxna. I dessa studier har använts c:a 3-5 mg/dag och de biverkningar som noterats har varit trötthet, yrsel och illamående men inte signifikant mer än vad som noterats i placebogrupperna (review/thesis Andersen LP 2015). I den RCT där 10 mg använts dagligen i 8 veckor noterades heller inga allvarliga biverkningar vilket också bekräftas av en toxikologisk dubbelblind RCT (Seabra et al 2000) som genomfördes där behandlingsgruppen erhöll 10 mg melatonin dagligen i 4 veckor och där ingen skillnad noterades mellan behandlade och placebogrupp. För att undersöka långtidseffekten vid högre doser gavs upp till 300 mg/dagligen upp till 2 år till patienter med ALS (Weishaupt et al 2006). Inga allvarliga biverkningar rapporterades, däremot fann man att cirkulerande serum protein carbonyls (en surrogat markör för oxidativ stress) vilken var förhöjd hos ALS patienterna, normaliserades under melatonin behandlingen. En analys av melatonin koncentrationen efter 2 månaders behandling visade att plasmanivåerna var kvar inom förväntade nivåer vilket indikerar att ingen ackumulation av melatonin skedde och inte heller någon ökad metabolism.

**Utvärdering av smärta**

Den vanligaste och mest utvärderade formen av smärtskattning är VAS skalan, där individen skattar på 10 cm lång linje intensiteten av smärtan. 0 motsvarar ingen smärta och 10 cm värsta tänkbara. Detta instrument fungerar bäst för akut smärta. För kronisk smärta som endometrios kan detta kombineras med livskvalitetsenkät, specifikt utvärderad för endometrios, EHP-30. Vid andra typer av kronisk smärta har ofta också använts Pain Catastrophizing Scale (PCS) där individen upplever olika känslor och tankar av hopplöshet, otålighet, rädsla och oro till följd av den kroniska smärtan. Detta instrument har utvärderats i många studier men ännu inte just specifikt för endometrios.

**Dosval**

För kvinnor med akut smärta så som dysmenorre förefaller det adekvat att använda 10 mg dagligen då den dosen visat god effekt på den akuta smärtan som utvärderats med VAS skala i tidigare studier och även visat signifikant minskad smärta hos kvinnor med endometrios. Det finns dock inga studier som utvärderat effekten av melatonin på just menssmärta. Teoretiskt är det möjligt att den analgetiska effekten utövas via opioid receptorn vid akut smärta . Detta är förmodligen en av verkningsmekanismerna även vid den kroniska smärtan men där kan det vara möjligt att melatonins antioxidativa och antiinflammatoriska effekt bidrar i högre utsträckning. Kvinnor med endometrios har till följd av endometrioslesioner i lilla bäckenet ofta andra associerade symtom som samlagssmärta, tarmtömningssmärta, tarmbesvär (Irritable bowel syndrome IBS). Vid detta tillstånd krävs sannolikt en högre dos vilket stöds av att dessa endometriosassocierade symtom fortfarande varit ganska uttalade när 10 mg använts (Schwertner et al). Djurstudier har visat en påtaglig dos-respons effekt på inducerade endometrioslesioner. Dessa lesioner minskade signifikant mer vid dosen 20mg/kg/dag jämfört med 10mg/kg/dag (Cetinkaya et al 2015). Då melatonin förefaller synnerligen atoxiskt har vi därför valt att undersöka effekten av 20 mg dagligen på smärta vid endometrios.

**Syfte**

Det aktuella projektet syftar till att finna en effektiv metod för smärtlindring, av menstruationsorsakad bäckensmärta, som också accepteras väl av kvinnan. Vi vill också utvärdera om smärtlindringsmetoden också medför en positiv effekt på det dagliga livet såsom förbättrad kognition eller upplevelse av livskvalitet, i jämförelse med placebo. För att få en uppfattning om en förbättrad sömnkvalitet bidrar till en eventuell effekt av livskvalitet eller kognition kommer vi också att använda en utvärderad enkät bestående av 3-7 frågor om sömnen.

**Frågeställningar**

Kan melatonin givet under menstruationsfasen minska smärtan hos kvinnor med svår mensvärk?

Har kvinnor med svår mensvärk nedsatt kognition under menstruationsfasen och kan denna nedsättning i så fall minskas under behandling med melatonin?

Kan kontinuerligt administrerad melatonin minska smärtan och förbättra livskvaliteten hos kvinnor med konstaterad endometrios?

Kan förbättrad sömn bidra till upplevd eventuellt förbättring av smärta och/eller livskvalitet?

**Primär utfallsvariabel**

Smärtskattning på en visuell analog skala (VAS). En minskning av maximal smärtskattning motsvarande minst 13 mm anses som signifikant

**Sekundära utfallsvariabler**

Behov av analgetika i tillägg till studieläkemedel, jämfört med placebo

Bedömning av kognition med melatonin jämfört med placebo (kvinnor med dysmenorre)

Bedömning av sömnkvalitet med melatonin jämfört med placebo (dysmenorre och endometrios)

Bedömning av livskvalitet med melatonin jämfört med placebo (kvinnor med endometrios)

Upplevelse av katastrofkänsla (Catastrophizing Pain Scale=CPS) för endometriosgruppen

Eventuella sidoeffekter av melatonin

Acceptans av melatonin

**Design**

Två delprojekt planeras.

1. Randomiserad placebokontrollerad jämförande studie (dysmenorre)
2. Randomiserad placebokontrollerad jämförande studie (endometrios)

**Metod**

1. 40 kvinnor med svår mensvärk (VAS > 6) kommer att rekryteras. Vid screening besöket får de besvara sömnkvalitetsenkäten ISI. De observeras under en menscykel där de rapporterar grad av smärta samt intag av analgetika under menstruationen. Därefter randomiseras deltagarna till melatonin 10 mg peroralt dagligen, eller placebo, under 7 dagar med start när menstruationen börjar. Läkemedlet intas till natten, vid sänggående, förutom den första dosen som intas så fort symtom på smärta uppträder (bolusdos). Ett kognitionstest (Cambridge Cognitive Battery Test) som testar minne, koncentration, uppmärksamhet, organisation) kommer att genomföras under menstruationsveckan dag 2 eller 3 i screeningscykel samt i behandlingscykel 2. Deltagarna ska dagligen föra dagbok över blödning, läkemedelsintag (inklusive annan analgetika),samt smärtupplevelse (VAS) och eventuella sidoeffekter. Analgetika förskrivs av studieansvariga läkare (Ibuprofen samt Paracetamol upp till rekommenderad högsta dos).
2. 40 kvinnor med diagnostiserad endometrios inkluderas i projektet. Vid screening besöket får de besvara sömnkvalitetsenkäten ISI. De kommer att observeras under 4 veckor där smärta och behov och intag av analgetika registreras. Därefter lottas de till daglig behandling med 20 mg melatonin, eller placebo, till natten under 8 veckor. Deltagarna kommer att utföra dagliga smärtskattningar (VAS skala) och rapportera allt intag av analgetika. I slutet av screeningmånaden respektive andra behandlingsmånaden utvärderas kvinnornas upplevelse av livskvalitet genom att besvara EHP-30 (QoL) som är utvärderad i tidigare studier av menstruationsbesvär samt Pain Catastrophizing Scale, PCS (som inte är validerad för endometrios).

   Efter avslutade studier kommer deltagarna i båda delprojekten att få fylla i ISI för andra gången samt en kort enkät (två frågor) om hur de upplevt behandlingen dvs är de nöjda och skulle de vilja fortsätta med studieläkemedlet.

**Inklusionskriterier**

**Studie 1.**

Kvinna, gott allmäntillstånd, över 18 år med regelbunden mens och svår mensvärk (VAS>6)

Villig att föra studiedagbok under hela studietiden (3 månader)

Talar och förstår svenska

Om annan läkemedelsbehandling ska denna ha pågått oförändrad i minst 3 månader

**Studie 2.**

Kvinna, gott allmäntillstånd, över 18 år med konstaterad endometrios. Måttliga till svåra bäckensmärtor (VAS≥4) sedan minst 6 månader

Villig att föra studiedagbok under hela studietiden (3 månader)

Talar och förstår svenska

Om annan läkemedelsbehandling ska denna ha pågått oförändrad i minst 3 månader

**Exklusionskriterier**

**Studie 1.**

<18 år
Oregelbunden mens (<21 eller >35 dagars cykler) eller amenorre

Kontinuerlig hormonbehandling som medför amenorre

Nedsatt lever eller njurfunktion i sjukhistorien

Konstaterad endometrios

Rökare

Ej svensktalande

Gravid

Nyinsatt läkemedelsbehandling, kortare än 3 månaders behandling

**Studie 2.**

<18 år

Nedsatt lever eller njurfunktion i sjukhistorien

Ej verifierad endometrios

Opioidbehandlad smärta

Rökare

Ej svensktalande

Gravid

Nyinsatt läkemedelsbehandling, kortare än 3 månaders behandling

**Studiepopulationens storlek**

Storleken på studiepopulationen baseras på tidigare studier av smärtpatienter där man beräknat att en skillnad i VAS-skalan på 13mm är en kliniskt relevant skillnad. För en power på 80% (signifikansnivå 0.05) skulle det behövas 15 individer i vardera gruppen, behandling respektive placebo. För att kompensera för bortfall under studiens grupp planerar vi att inkludera 40 kvinnor som randomiseras till melatonin eller placebo.

**Urval**

Deltagarna kommer att tillfrågas om deltagande när de söker på vår gynekologiska mottagning, på ungdomsmottagning eller om de själva anmäler intresse. Information om studien kommer att anslås på sjukhusets anslagstavlor. Brukarorganisationer (för endometrios) kommer också att informeras om projektet.

**Informerat samtycke**

Den som anmält intresse för deltagande informeras såväl muntligt som skriftligt av studieansvariga (sköterska och läkare). I samband med besök på forskningsenheten inhämtar forskningsansvarig läkare skriftligt samtycke om kvinnan accepterar deltagande.

**Statement of compliance**

Studien kommer att genomföras enligt protokollet och gällande regelverket, LVFS 2011:19, ICH GCP och senaste versionen av Helsingforsdeklarationen.

**Väsentlig ändring**

Väsentliga ändringar i protokollet (LVFS 2011:19,7 kap) måste godkännas av Läkemedelsverket och Etikprövningsnämnd innan de får implementeras i prövningen.

**Detaljerad plan för studiedeltagarna**

**Delstudie 1 (dysmenorre)**

Efter att samtycke till deltagande erhållits får studiedeltagarna monitorera en menstruationscykel med hjälp av studiedagbok. Denna utdelas vid det första screeningbesöket. Här registreras dagligen blödningar, smärta (skattas på VAS skala) samt intag av analgetika (typ och dos samt tidpunkt). Efter denna inledande cykel, under förutsättning att inklusionskritererna fortfarande är uppfyllda, randomiseras deltagaren vid det första återbesöket till inledande behandling med aktivt läkemedel eller placebo. Studieläkemedel för två menscykler samt recept på analgetika utdelas av forskningssköterska. Sömnenkät besvaras (Insomnia severity index) Deltagaren fortsätter fylla i studiedagboken dagligen.

Under menstruationsveckan, cykeldag 2-3, i screeningcykeln kommer deltagaren på nästa återbesök för att genomföra ett första kognitionstest. Detta utförs tillsammans med någon av studieansvariga och beräknas ta c:a 30 minuter.

Under menstruationsveckan, cykel dag 2-3, i behandlingscykel två kommer deltagaren på återbesök för att genomföra det andra kognitionstestet.

Det sista återbesöket sker när deltagaren genomfört två behandlingscykler och innebär att hon återlämnar övertaliga läkemedel samt förpackningar, studiedagbok och ombeds besvara sömnenkät ånyo samt 4 frågor om acceptansen av behandlingen. En graviditetstest tas även vid detta tillfälle. Totalt beräknas fem besök under studieperioden som totalt omfattar c:a tre månader.

**Delstudie 2 (endometrios)**

Efter att samtycke till deltagande erhållits får studiedeltagarna monitorera en fyra veckorsperiod (eller menstruationscykel för de som inte är amenorroiska) med hjälp av studiedagbok. Denna utdelas vid det första screeningbesöket. Här registreras dagligen blödningar, smärta (skattas på VAS skala) samt intag av analgetika (typ och dos samt tidpunkt). Efter denna inledande cykel, under förutsättning att inklusionskritererna fortfarande är uppfyllda, randomiseras deltagaren vid det första återbesöket till inledande behandling med aktivt läkemedel eller placebo. Studieläkemedel för två månader utdelas och analgetika förskrivs. Formulären EHP-30, ISI, PCS 1 genomförs och deltagaren fortsätter fylla i studiedagboken dagligen.

Studiedeltagaren intar den första dosen av studieläkemedlet på första dagen i behandlingsmånad 1. Därefter tas en dos vid sänggåendet under två månader.

Under sista veckan i behandlingsmånad två sker nästa återbesök då deltagaren fyller återigen i formulären PCS 1 EHP-30 (Quality of Life).

Det sista återbesöket sker när deltagaren genomfört fyra behandlingscykler och innebär att hon återlämnar övertaliga läkemedel samt förpackningar, studiedagbok och ombeds besvara sömnenkäten och 4 frågor om acceptansen av behandlingen. En graviditetstest tas även vid detta tillfälle. Totalt beräknas 4 besök under studieperioden som totalt omfattar c:a tre månader.

**Deltagarlogg**

Alla som genomgår screening kommer att registreras i screening logg och alla som inkluderas kommer att registreras i studielogg.

**Behandling – Studieläkemedel**

Läkemedlet som ska studeras är melatonin (APL) se bifogad resume.

Det är kapslar på 5 mg och dosen som används i delstudie 1 är 10 mg under menstruationsperioden. I delstudie 2 är det 20 mg dagligen. Dessutom kommer deltagarna att kunna inta paracetamol och/eller ibuprofen som tilläggsanalgetika om det behövs. Maximala dygnsdoser av paracetamol och ibuprofen överensstämmer med rekommendation enligt FASS. Samtliga läkemedel mot smärta utdelas från forskningsenheten. Kapslarna lagras och märks upp av APL inför utdelning till deltagarna och registreras i en logg på enheten. Batch nummer och utgångsdatum står på samtliga förpackningar samt studiedeltagarens studie ID och ansvarig läkare (se märkningsförslag).

**Läkemedelshantering**

Förpackning och märkning av läkemedel kommer att utföras vid APL

**Randomisering**

Randomisering kommer att ske med hjälp av webbaserat randomiseringsprogram.

**Analys**

Studiepopulationen kommer att analyseras med deskriptiv statistik. Skillnaderna i smärtupplevelse kommer att analyseras med Mixed models eller repeated ANOVA. Skillnaderna i resultaten på enkäter som ingår kommer att analyseras med Wilcoxon test
Analyser av bortfall i studien kommer att beskrivas i detalj innan studien avslutas dvs innan databasen låses och avblindas. Protokollet kommer att uppdateras med denna information.

**Avvikande händelser (****Adverse events=AE)**

Alla medicinskt oväntade händelser som drabbar en studiedeltagare som erhåller studieläkemedel definieras som Avvikande händelse och ska rapporteras. Detta oavsett om det har ett tydligt orsakssamband med läkemedlet eller inte. Det kan vara ett symtom, ett kliniskt fynd eller ett laboratoriesvar. AE ska bedömas avseende intensitet och klassifieras som mild (acceptabla för deltagaren), måttlig (störande för daglig aktivitet) eller allvarlig(oacceptabel).

En AE ska också bedömas avseende samband, kausalitet som otänkbar, möjlig eller trolig

**Avvikande reaktion (Adverse Reactions)**

Varje AE ska klassifieras som relaterad eller inte relaterad till studieläkemedlet. En AR är en skadlig och oväntad reaktion som högst troligt framkallas av läkemedlet.

**Allvarlig avvikande händelse (Serious adverse events=SAE)**

Allvarlig avvikande händelse, SAE, definieras som en AE som :

-resulterar i död

-är livshotande

-kräver sjukhusvård

-medför uppenbar påverkan på allmäntillståndet

-medför medfödd missbildning

-andra medicinskt allvarliga händelser

SAE ska rapporteras till sponsor inom 24 timmar efter att det kommit till studieansvarigas kännedom. Det ska ske på särskilt SAE formulär (finns i CRF)

Alla SAE ska årligen rapporteras till Läkemedelsverket och etiknämnden

**Misstänkt oväntad SAR (SUSAR)**

En SAE som är troligt relaterad till studieläkemedlet och oväntad (dvs inte nämnd i tidigare studier eller investigators brochure) kallas Suspected Unexpected serious Adverse Reaction (SUSAR) och anmäles av huvudprövare (sponsor) till etikprövningsnämnden (per post inom 7 dagar) och till Läkemedelsverket via Eudravigilandssystemet inom 15 dagar. Huvudprövare (sponsor) ansvarar för att informera övriga studieansvariga. Studien kommer enbart att pågå vid ett center.

**Risk/nytta värdering**

Om effekten är så god som vi hoppas på kommer de som får aktivt läkemedel att uppleva en klar förbättring av sitt tillstånd. Detta skulle i så fall medföra ett välkommet tillskott i behandlingsarsenalen för bäckensmärtor hos kvinnor. Risken kvinnorna utsätts för är att de under en period kan få verkningslös behandling eller behandling som inte ger tillräcklig smärtlindrande. Om smärtan inte lindras får deltagarna använda alternativa smärtlindrande läkemedel. Melatonin har inte visats vara associerat med några allvarliga bieffekter. Preparatet har använts länge och i olika doser varav även i doser betydligt högre än vad vi planerar. Behandlingsperioderna har också varit betydligt längre än vad vi avser i projektet

**Kriterier för avbrott i studien**

Deltagarna kan när som helst och utan att uppge skäl avbryta sitt deltagande. Studieansvariga kan också avbryta deltagare från att delta i studien om det uppstår t.ex graviditet eller en situation där deltagarens säkerhet inte längre kan garanteras. Studieansvariga kan också avbryta deltagande om studiedeltagare inte längre uppfyller inklusionskriterier eller om någon komplikation inträffat (AE) som medför en negativ effekt på deltagaren enligt prövarens bedömning

Om detta inträffar kommer en förklaring till avbrytandet att dokumenteras och en utvärdering ändå att göras fram till den tidpunkt då deltagandet avslutas. Alla deltagare som avslutar eller avslutas i förtid kommer att inkluderas i den slutliga sammanställningen.

**Brytning av kodning**

I händelse av SUSAR (se avsnitt ovan) kommer forskningsdeltagarens kod för blindning av behandling att brytas av studieansvarig huvudprövare (sponsor).

**Kvalitetskontroll**

Studieansvarig ansvarar för att all data förvaras på ett säkert sätt. CRF (Case Report Form) fylls i av studieansvarig sköterska och läkare på enheten (Södersjukhuset).

Forskningssköterska från Linköpings Universitetssjukhus kommer att genomföra monitorering av all data under studiens gång.

**Källdata**

Case Report Form (CRF) och medicinska journaler utgör källdata. I CRF finns de huvudsakliga uppgifterna om studien så som intag av läkemedel, menstruationsdata och smärtdagbok samt deskriptiv information som ålder, längd och vikt. I den elektroniska patientjournalen finns uppgifter om patientens aktuella hälsotillstånd, laboratoriesvar samt uppgift om information och inhämtat samtycke avseende prövningen. Som källdata räknas också de enkäter som ingår i projektet (QoL, PCS och Cambridge Cognitive Battery Test).

**Tillgång till källdata**

Monitor (och personal från regulatorisk myndighet) garanteras tillgång till källdata och till prövningen tillhörande dokument, som är nödvändiga för att kunna utföra monitorering och inspektion. Monitorering och inspektion kan utföras när som helst under eller efter prövningen.

**Förvaring av källdata**

CRF och övriga källdata (se ovan) kommer att förvaras inlåst 10 år efter avslutande av studie. Journaler (datajournal) följer sjukhusets regler för arkivering som dock inte understiger 10 år.

**Tidsplan**

Rekrytering och studiestart planeras till tidigast november 2018 och beräknas pågå 18 månader. Därefter analys och bearbetning av data under 2020.

**Referenser:**

Andersch B, Milsom I. An epidemiologic study of young women with dysmenorrheal Am J Obstet Gynecol 1982;144:655-660

Andersen LP. The analgesic effects of exogenous melatonin in humans
Thesis Dan Med J 2016;63(10):B5289

Bale P, Davis J. Effect on menstruation and contraceptive pill on the performance of physical education students Br J Sports Med 1983;17:46-50

Bourdel N, Alves J, Pickering G, Ramilo I, Roman H, Canis M. Systematic review of endometriosis pain assessment: how to choose a scale? Hum Reprod Update. 2015 Jan-Feb;21(1):136-52

Brown J, Crawford TJ, Allen C, Hopewell S, Prentice A. Nonsteroidal anti-inflammatory drugs for pain in women with endometriosis. Cochrane Database
Syst Rev. 2017 Jan 23;1:CD004753. doi: 10.1002/14651858.CD004753.pub4.

Chen WW, Zhang X, Huang WJ. Pain control by melatonin: Physiological and pharmacological effects (Review) Exp Ther Medicine 2016;12:1963-68

Clark TJ, Khan KS, Foon R, Pattison H, Bryan S, Gupta JK. Quality of life instruments in studies of menorrhagia: a systematic review. Eur J Obstet Gynecol Reprod Biol 2002;104:96-104

Facchin F, Barbara G, Saita E, Mosconi P, Roberto A, Fedele L, Vercellini P. Impact of endometriosis on quality of life and mental health: pelvic pain makes the difference J Psychosom Obstet Gynaecol, 2015;36(4):135-141

Güney M, Oral B, Karahan N, Mungan T.Regression of endometrial explants in a rat model of endometriosis treated with melatonin.Fertil Steril. 2008 Apr;89(4):934-42

Harel Z. Dysmenorrhea in adolescents. Ann N Y Acad Sci. 2008;1135:185-95. doi: 10.1196/annals.1429.007.

Hillen TI1, Grbavac SL, Johnston PJ, Straton JA, Keogh JM. Primary dysmenorrhea in young Western Australian women: prevalence, impact, and knowledge of treatment. J Adolesc Health. 1999 Jul;25(1):40-5.

Laufer MR, Goitein L, Bush M, Cramer DW, Emans SJ. Prevalence of endometriosis in adolescent girls with chronic pelvic pain not responding to conventional therapy.J Pediatr Adolesc Gynecol. 1997 Nov;10(4):199-202.

Márki G, Bokor A, Rigó J, Rigó A. Physical pain and emotion regulation as the main predictive factors of health-related quality of life in women living with endometriosis. Hum Reprod. 2017 May 8:1-7. doi: 10.1093/humrep/dex091.

Saha R, Kuja-Halkola R, Tornvall P, Marions L. Reproductive and Lifestyle Factors Associated with Endometriosis in a Large Cross-Sectional Population Sample. J Womens Health (Larchmt). 2016 Sep 15

Saha R, Pettersson HJ, Svedberg P, Olovsson M, Bergqvist A, Marions L, Tornvall P, Kuja-Halkola R. Heritability of endometriosis. Fertil Steril. 2015 Oct;104(4):947-5

Schwertner A, Conceição Dos Santos CC, Costa GD, Deitos A, de Souza A, de Souza IC, Torres IL, da Cunha Filho JS, Caumo W. Efficacy of melatonin in the treatment of endometriosis: a phase II, randomized, double-blind, placebo-controlled trial. Pain. 2013 Jun;154(6):874-81. doi: 10.1016/j.pain.2013.02.025

Seabra MLV, Bignotto M, Pinto LR, Tufik S. Randomized, double-blind clinical trial, controlled with placebo, of the toxicology of chronic melatonin treatment J. Pineal Res. 2000;29:193-200

Suvitie PA, Hallamaa MK, Matomäki JM, Mäkinen JI, Perheentupa AH. Prevalence of Pain Symptoms Suggestive of Endometriosis Among Finnish Adolescent Girls (TEENMAPS Study). J Pediatr Adolesc Gynecol. 2016 Apr;29(2):97-103.

MJL Sullivan, SR Bishop, J Pivik. The pain catastrophizing scale: development and validation. Psychological assessment, 1995 - psycnet.apa.org

Todd KH, Funk KG, Funk JP, Bonacci R. Clinical significance of reported changes in pain severity. Ann Emerg Med. 1996 Apr;27(4):485-9.

Weishaupt JH, Bartels C, Pölking E, Dietrich J, Rohde G, Poeggeler B, Mertens N, Sperling S, Bohn M, Huther G, Schneider A, Bach A, Siren AL, Hardeland R, Bähr M, Nave KA, Ehrenreich H. Reduced oxidative damage in ALS by high-dose enteral melatonin treatment. J Pineal Res. 2006;41:313-323

Yilmaz B, Kilic S, Aksakal O, Ertas IE, Tanrisever GG, Aksoy Y, Lortlar N, Kelekci S, Gungor T. Melatonin causes regression of endometriostic implants in rats by modulating angiogenesis, tissue levels of antioxidants and matrix metalloproteinases Arch Gynecol Obstet 2015;292:209-216

Yildirim G, Attar R, Ozkan F, Kumbak B, Ficicioglu C, Yesildaglar N. The effects of letrozole and melatonin on surgically induced endometriosis in a rat model: a preliminary study Fertil Steril 2010;93:1787-1792
