# Supplementary material for: Adjuvant use of melatonin for pain management in endometriosis-associated pelvic pain—A randomized double-blinded, placebo-controlled trial
Source: PLoS One. 2023 Jun 2;18(6):e0286182. doi: 10.1371/journal.pone.0286182 (PMC10237656; doi:10.1371/journal.pone.0286182)
Supplement: Attachment — Submitted filename: Response to reviewers.docx [file pone.0286182.s005.docx]

PONE-D-22-34783

Adjuvant use of melatonin for pain management in endometriosis-associated pelvic pain - a randomized double-blinded, placebo-controlled trial

PLOS ONE

Dear Dr. Söderman,

Thank you for submitting your manuscript to PLOS ONE. After careful consideration, we feel that it has merit but does not fully meet PLOS ONE’s publication criteria as it currently stands. Therefore, we invite you to submit a revised version of the manuscript that addresses the points raised during the review process.

ACADEMIC EDITOR:

Please respond to all reviewers comments clearly point by point

Please submit your revised manuscript by Mar 24 2023 11:59PM. If you will need more time than this to complete your revisions, please reply to this message or contact the journal office at [plosone@plos.org](mailto:plosone@plos.org). When you're ready to submit your revision, log on to <https://www.editorialmanager.com/pone/> and select the 'Submissions Needing Revision' folder to locate your manuscript file.

Please include the following items when submitting your revised manuscript:

- A rebuttal letter that responds to each point raised by the academic editor and reviewer(s). You should upload this letter as a separate file labeled 'Response to Reviewers'.
- A marked-up copy of your manuscript that highlights changes made to the original version. You should upload this as a separate file labeled 'Revised Manuscript with Track Changes'.
- An unmarked version of your revised paper without tracked changes. You should upload this as a separate file labeled 'Manuscript'.

If you would like to make changes to your financial disclosure, please include your updated statement in your cover letter. Guidelines for resubmitting your figure files are available below the reviewer comments at the end of this letter.

If applicable, we recommend that you deposit your laboratory protocols in protocols.io to enhance the reproducibility of your results. Protocols.io assigns your protocol its own identifier (DOI) so that it can be cited independently in the future. For instructions see: [https://journals.plos.org/plosone/s/submission-guidelines#loc-laboratory-protocols](https://eur01.safelinks.protection.outlook.com/?url=https%3A%2F%2Fjournals.plos.org%2Fplosone%2Fs%2Fsubmission-guidelines%23loc-laboratory-protocols&data=05%7C01%7Clisa.soderman%40ki.se%7C4c275ecc93af4d56d96008db0931bf23%7Cbff7eef1cf4b4f32be3da1dda043c05d%7C0%7C0%7C638113881646588552%7CUnknown%7CTWFpbGZsb3d8eyJWIjoiMC4wLjAwMDAiLCJQIjoiV2luMzIiLCJBTiI6Ik1haWwiLCJXVCI6Mn0%3D%7C3000%7C%7C%7C&sdata=p5ReyIgfABTy%2FPCxCi8Fj2TQf3jMu%2BjZtwGExB7%2BUtY%3D&reserved=0). Additionally, PLOS ONE offers an option for publishing peer-reviewed Lab Protocol articles, which describe protocols hosted on protocols.io. Read more information on sharing protocols at [https://plos.org/protocols?utm_medium=editorial-email&utm_source=authorletters&utm_campaign=protocols](https://eur01.safelinks.protection.outlook.com/?url=https%3A%2F%2Fplos.org%2Fprotocols%3Futm_medium%3Deditorial-email%26utm_source%3Dauthorletters%26utm_campaign%3Dprotocols&data=05%7C01%7Clisa.soderman%40ki.se%7C4c275ecc93af4d56d96008db0931bf23%7Cbff7eef1cf4b4f32be3da1dda043c05d%7C0%7C0%7C638113881646588552%7CUnknown%7CTWFpbGZsb3d8eyJWIjoiMC4wLjAwMDAiLCJQIjoiV2luMzIiLCJBTiI6Ik1haWwiLCJXVCI6Mn0%3D%7C3000%7C%7C%7C&sdata=vzTiJ1nv9BS1O%2FetReyazaQeZvd%2BrWlQzNVA8AJ%2FZc4%3D&reserved=0).

We look forward to receiving your revised manuscript.

Kind regards,

Ahmed Mohamed Maged, MD
Academic Editor
PLOS ONE

Journal Requirements:

When submitting your revision, we need you to address these additional requirements.

1. Please ensure that your manuscript meets PLOS ONE's style requirements, including those for file naming. The PLOS ONE style templates can be found at 
<https://journals.plos.org/plosone/s/file?id=wjVg/PLOSOne_formatting_sample_main_body.pdf> and 
<https://journals.plos.org/plosone/s/file?id=ba62/PLOSOne_formatting_sample_title_authors_affiliations.pdf>

- Done.

2. We noticed you have some minor occurrence of overlapping text with the following previous publication(s), which needs to be addressed:

- 10.1007/s00228-021-03234-6. Epub 2021 Oct 20.

In your revision ensure you cite all your sources (including your own works), and quote or rephrase any duplicated text outside the methods section. Further consideration is dependent on these concerns being addressed.

- I have addressed and rephrased the overlappning text.

3. We note that the grant information you provided in the ‘Funding Information’ and ‘Financial Disclosure’ sections do not match.

When you resubmit, please ensure that you provide the correct grant numbers for the awards you received for your study in the ‘Funding Information’ section.

-So sorry about that! I verified it is correct.

4. Thank you for stating the following in the Competing Interests section:

"LS, LM, YB, HJP have no conflict of interest. ME reports employment and stock ownership with SOBI AB, and previously with Vifor Pharma AB."

Please confirm that this does not alter your adherence to all PLOS ONE policies on sharing data and materials, by including the following statement: "This does not alter our adherence to  PLOS ONE policies on sharing data and materials.” (as detailed online in our guide for authors <http://journals.plos.org/plosone/s/competing-interests>).  If there are restrictions on sharing of data and/or materials, please state these. Please note that we cannot proceed with consideration of your article until this information has been declared.

Please include your updated Competing Interests statement in your cover letter; we will change the online submission form on your behalf.

- The statement is included in the cover letter

5. In your Data Availability statement, you have not specified where the minimal data set underlying the results described in your manuscript can be found. PLOS defines a study's minimal data set as the underlying data used to reach the conclusions drawn in the manuscript and any additional data required to replicate the reported study findings in their entirety. All PLOS journals require that the minimal data set be made fully available. For more information about our data policy, please see <http://journals.plos.org/plosone/s/data-availability>.

Upon re-submitting your revised manuscript, please upload your study’s minimal underlying data set as either Supporting Information files or to a stable, public repository and include the relevant URLs, DOIs, or accession numbers within your revised cover letter. For a list of acceptable repositories, please see <http://journals.plos.org/plosone/s/data-availability#loc-recommended-repositories>. Any potentially identifying patient information must be fully anonymized.

Important: If there are ethical or legal restrictions to sharing your data publicly, please explain these restrictions in detail. Please see our guidelines for more information on what we consider unacceptable restrictions to publicly sharing data: <http://journals.plos.org/plosone/s/data-availability#loc-unacceptable-data-access-restrictions>. Note that it is not acceptable for the authors to be the sole named individuals responsible for ensuring data access.

We will update your Data Availability statement to reflect the information you provide in your cover letter.

6. Please review your reference list to ensure that it is complete and correct. If you have cited papers that have been retracted, please include the rationale for doing so in the manuscript text, or remove these references and replace them with relevant current references. Any changes to the reference list should be mentioned in the rebuttal letter that accompanies your revised manuscript. If you need to cite a retracted article, indicate the article’s retracted status in the References list and also include a citation and full reference for the retraction notice.

- I have reviewed the reference list.

Additional Editor Comments:

Please respond to all reviewers comments

[Note: HTML markup is below. Please do not edit.]

Reviewers' comments:

Reviewer's Responses to Questions

**Comments to the Author**

1. Is the manuscript technically sound, and do the data support the conclusions?

The manuscript must describe a technically sound piece of scientific research with data that supports the conclusions. Experiments must have been conducted rigorously, with appropriate controls, replication, and sample sizes. The conclusions must be drawn appropriately based on the data presented.

Reviewer #1: Yes

Reviewer #2: Yes

Reviewer #3: Yes

2. Has the statistical analysis been performed appropriately and rigorously?

Reviewer #1: Yes

Reviewer #2: Yes

Reviewer #3: Yes

3. Have the authors made all data underlying the findings in their manuscript fully available?

The [PLOS Data policy](https://eur01.safelinks.protection.outlook.com/?url=http%3A%2F%2Fwww.plosone.org%2Fstatic%2Fpolicies.action%23sharing&data=05%7C01%7Clisa.soderman%40ki.se%7C4c275ecc93af4d56d96008db0931bf23%7Cbff7eef1cf4b4f32be3da1dda043c05d%7C0%7C0%7C638113881646588552%7CUnknown%7CTWFpbGZsb3d8eyJWIjoiMC4wLjAwMDAiLCJQIjoiV2luMzIiLCJBTiI6Ik1haWwiLCJXVCI6Mn0%3D%7C3000%7C%7C%7C&sdata=vGMKeF1ZIdmFuqc4PAVm5P8JUBems%2Ff1BzWHXuH0Ujc%3D&reserved=0) requires authors to make all data underlying the findings described in their manuscript fully available without restriction, with rare exception (please refer to the Data Availability Statement in the manuscript PDF file). The data should be provided as part of the manuscript or its supporting information, or deposited to a public repository. For example, in addition to summary statistics, the data points behind means, medians and variance measures should be available. If there are restrictions on publicly sharing data—e.g. participant privacy or use of data from a third party—those must be specified.

Reviewer #1: No – DOI is now included

Reviewer #2: No - DOI is now included

Reviewer #3: Yes

4. Is the manuscript presented in an intelligible fashion and written in standard English?

PLOS ONE does not copyedit accepted manuscripts, so the language in submitted articles must be clear, correct, and unambiguous. Any typographical or grammatical errors should be corrected at revision, so please note any specific errors here.

Reviewer #1: Yes

Reviewer #2: Yes

Reviewer #3: Yes

5. Review Comments to the Author

Please use the space provided to explain your answers to the questions above. You may also include additional comments for the author, including concerns about dual publication, research ethics, or publication ethics. (Please upload your review as an attachment if it exceeds 20,000 characters)

**Reviewer #1**: A randomized-controlled clinical trial compared the level of pain between melatonin treatment and placebo in women with endometriosis-associated pain. No statistical differences in pain levels between the arms were observed.

Minor revisions:
1- Line 113: State the statistical testing method that achieves 80% power. If an estimate of the standard deviation was used in the power calculation, state it.

-We have clarified the power calculation and specified the assumed standard deviation that we used. The power calculation was based on independent t-test and we have clarified this by adding a reference to the program that we used for the calculation.

2- Line 127: The standard statistical term for average is mean. – Changed

3- Table 1: Indicate if the summarized values in the table are means and standard deviations. If data is normally distributed, means and standard deviations are appropriate. However, if the data is non-normally distributed median, first and third quartiles are typically provided. – We specified that the numbers are mean values.

4- Line 148: Indicate the statistical testing method(s) used to conclude that the variables in table 1 were similar/different when comparing the groups. – It is now specified in the statistics section, we used independent t -test.

5- Line 149-156: In addition to the frequencies, provide the corresponding percentages. – Added

6- Line 161: Consider rephrasing the following sentence. Both the terms differences and similar were used which is confusing to the reader. “No statistically significant interaction between time and treatment group was seen, which imply that differences between the groups were similar for all time points.” – Rephrased to a, hopefully, more comprehensive phrase.

7- Line 163: State the percent with insomnia in each arm. – Added.

8- Define the abbreviation SD at its first occurrence. - Added.

**Reviewer #2**: This is a well thought out experiment. I agree with the authors that the context of the experiment allows for high internal validity. It is clear that the authors are aware of both the strengths and the weaknesses of the experiment.

- I think implementing a stratified random sampling to account for previous hormone therapy users and any other important confounders, ie, the number of amenorrhea patients recruited after the exclusion criteria was relaxed, would benefit the analyses and interpretation of the data.

-We agree, a sensitivity analysis stratified by hormonal treatment is added as a supplementary table. However due to the small number of observations it is hard to interpret.

- It also would be helpful to know when the patients in both arms conducted their self-assessments, since it is stated that the half life of melatonin is somewhere between 20 and 60 minutes.

– We clarified that the self-assessment was also made at bedtime.

- For that matter, knowing the within-patient variance of documentation of self-assessments would be beneficial to the strength of the study.

- We agree but unfortunately the data does not provide that information.

- The authors need to better define and describe the interaction terms used in the regression models. And disclosure of the model equation, including estimates and SEs, should be done before final acceptance.

-We agree. In the statistical method section, we have now clarified which model we used in order to make inferens about the treatment difference between the groups. And which variables that were used in the models, when the interactions were added.

It is my judgment that this experiment adds value to the scientific conversation regarding the association between melatonin and EAPP.

**Reviewer #3:** Thank you for giving me the opportunity to review this interesting RCT "Adjuvant use of melatonin for pain management in endometriosis-associated pelvic pain".

However, I have some comments and recommendations:

Title:- Is appropriate for the content of the article. – Thank you

Abstract:- Represents a suitable summary of the study. -Thank you

Introduction:- Emphasize the mechanism of action of melatonin. – We elaborated on the role of melatonin

Methods:- Mention method of randomization. – the very last section of method explains the randomization

Results:- Mention p values for baseline data. - Added

Discussion:- Emphasize the differences from other similar studies. - We clarified some differences

Conclusions:- Are justified on the basis of results of the study. ­– Thank you

References:- Follow the format of the journal. – We changed the format accordingly

General:- Revise language, grammar and syntax. - done

6. PLOS authors have the option to publish the peer review history of their article ([what does this mean?](https://eur01.safelinks.protection.outlook.com/?url=https%3A%2F%2Fjournals.plos.org%2Fplosone%2Fs%2Feditorial-and-peer-review-process%23loc-peer-review-history&data=05%7C01%7Clisa.soderman%40ki.se%7C4c275ecc93af4d56d96008db0931bf23%7Cbff7eef1cf4b4f32be3da1dda043c05d%7C0%7C0%7C638113881646588552%7CUnknown%7CTWFpbGZsb3d8eyJWIjoiMC4wLjAwMDAiLCJQIjoiV2luMzIiLCJBTiI6Ik1haWwiLCJXVCI6Mn0%3D%7C3000%7C%7C%7C&sdata=gsuDrGnB7FkLA3efmy0RMzlsdWQDgnXT%2FaQzbXRM4y4%3D&reserved=0)). If published, this will include your full peer review and any attached files.


If you choose “no”, your identity will remain anonymous but your review may still be made public.


**Do you want your identity to be public for this peer review?** For information about this choice, including consent withdrawal, please see our [Privacy Policy](https://eur01.safelinks.protection.outlook.com/?url=https%3A%2F%2Fwww.plos.org%2Fprivacy-policy&data=05%7C01%7Clisa.soderman%40ki.se%7C4c275ecc93af4d56d96008db0931bf23%7Cbff7eef1cf4b4f32be3da1dda043c05d%7C0%7C0%7C638113881646588552%7CUnknown%7CTWFpbGZsb3d8eyJWIjoiMC4wLjAwMDAiLCJQIjoiV2luMzIiLCJBTiI6Ik1haWwiLCJXVCI6Mn0%3D%7C3000%7C%7C%7C&sdata=tJpP7BZ7ps%2Fw6aelQ6ax3o%2FjqWt2EP565BGy91Ob6Fo%3D&reserved=0).

Reviewer #1: No

Reviewer #2: **Yes:** Thomas Falconer

Reviewer #3: No

[NOTE: If reviewer comments were submitted as an attachment file, they will be attached to this email and accessible via the submission site. Please log into your account, locate the manuscript record, and check for the action link "View Attachments". If this link does not appear, there are no attachment files.]

While revising your submission, please upload your figure files to the Preflight Analysis and Conversion Engine (PACE) digital diagnostic tool, [https://pacev2.apexcovantage.com/](https://eur01.safelinks.protection.outlook.com/?url=https%3A%2F%2Fpacev2.apexcovantage.com%2F&data=05%7C01%7Clisa.soderman%40ki.se%7C4c275ecc93af4d56d96008db0931bf23%7Cbff7eef1cf4b4f32be3da1dda043c05d%7C0%7C0%7C638113881646588552%7CUnknown%7CTWFpbGZsb3d8eyJWIjoiMC4wLjAwMDAiLCJQIjoiV2luMzIiLCJBTiI6Ik1haWwiLCJXVCI6Mn0%3D%7C3000%7C%7C%7C&sdata=sMpx0FmDoz%2Fd8mx%2FhSwsTo1aRtSmnwKgR6FuBOE%2BIk8%3D&reserved=0). PACE helps ensure that figures meet PLOS requirements. To use PACE, you must first register as a user. Registration is free. Then, login and navigate to the UPLOAD tab, where you will find detailed instructions on how to use the tool. If you encounter any issues or have any questions when using PACE, please email PLOS at [figures@plos.org](mailto:figures@plos.org). Please note that Supporting Information files do not need this step.

*In compliance with data protection regulations, you may request that we remove your personal registration details at any time.* [*(Remove my information/details)*](https://eur01.safelinks.protection.outlook.com/?url=https%3A%2F%2Fwww.editorialmanager.com%2Fpone%2Flogin.asp%3Fa%3Dr&data=05%7C01%7Clisa.soderman%40ki.se%7C4c275ecc93af4d56d96008db0931bf23%7Cbff7eef1cf4b4f32be3da1dda043c05d%7C0%7C0%7C638113881646588552%7CUnknown%7CTWFpbGZsb3d8eyJWIjoiMC4wLjAwMDAiLCJQIjoiV2luMzIiLCJBTiI6Ik1haWwiLCJXVCI6Mn0%3D%7C3000%7C%7C%7C&sdata=YmGqWxdd%2B2shSbQCF22DZfXv%2Fu0ldhjpXnxIWTlmSIc%3D&reserved=0)*. Please contact the publication office if you have any questions.*
