# Supplementary material for: Adjuvant use of melatonin for pain management in endometriosis-associated pelvic pain—A randomized double-blinded, placebo-controlled trial
Source: PLoS One. 2023 Jun 2;18(6):e0286182. doi: 10.1371/journal.pone.0286182 (PMC10237656; doi:10.1371/journal.pone.0286182)
Supplement: Attachment — Submitted filename: Response to reviewers v2.docx [file pone.0286182.s006.docx]

Journal Requirements:

The reference list is revised and is accurate. No changes have been made.

Reviewer #1: Minor revision:
1- Abstract: Include the corresponding standard deviations for 2.9 and 3.3. - Included

2- Line 118: State that the independent t-test was the statistical method used for estimating the power of the study. – the addition is made.

3- Line 155: For improved clarity consider replacing the sentence with the following. "The outcomes are presented as means and SD of the screen cycle." – I added SD.
